# Around the Hospitals

**Published:** 1896-07-04

**Authors:** 


### AROUND THE HOSPITALS.

[Notice to the Managers of Institutions.—Special space is now reserved for the insertion of notices of hospital meetings and festivals. The Editor requests that all notices may reach The Hospital Office, 428, Strand, at least one week before the date of each meeting.]

**Middlesex Hospital.**—The year 1895, during which this hospital completed its 150th anniversary, was one of great activity, and an increase of work done is to be noted in all departments. Thus the number of patients treated was 46,326, or 4,000 more than have ever before been recorded; the number of days' treatment given in the in-patient department has increased by rather more than 10 per cent. when compared with 1894 (the actual figures being 97,844 in 1895 and 88,802 for the year 1894); 300 patients were sent to various convalescent homes through the agency of the Samaritan Fund; a new convalescent home is being erected at Clacton-on-Sea, and will probably be ready for occupation by July 1st next; the new operating theatre was completed; and £9,300 out of the £12,000 estimated to be required was collected for the new cancer wing. No mean record for one year's work, the net result of which has been an increased expenditure of some £6,400, most of which may be attributed to building improvements. Most of the other items of expenditure only show increases which might be expected from the larger number of patients treated, the chief exception being an increase of some £600, due to a more elaborate cleaning being given in 1895 than was thought necessary in 1894, seeing that the hospital premises had been closed and completely overhauled in 1893. An interesting point in this connection is the fact that the annual cleaning now represents a much smaller outlay than used to be incurred before the introduction of electric light. Comparing the income with the expenditure, we find that the ordinary income was only £20,233, against an ordinary expenditure of £31,132, or a deficiency for the year of £10,899, against a deficiency in the same items in 1894 of £12,789. In both years over £12,000 was received in legacies to the general account, and by utilising these only a small deficit on the ordinary expenditure was shown for 1894, and a surplus of about £1,400 for 1895. Two contributions, amounting together to £3,400, were received for the Permanent Endowment Fund, and both were invested. One of these, a donation of £3,150, was given by Mr. Ludwig Neumann to be applied to the endowment of a bed in the Helena Ward, a cot in the Percy Ward, and a cot in the Princess May Ward. Seventy-seven male and 142 female cancer cases were admitted as in-patients during the year under review. The average number of beds constantly occupied during the whole year was 270, the average over the 51 days during which the annual cleaning was in progress being 220, and the average over the remainder of the year 279. The cost per occupied bed the hospital authorities calculate was £102 17s. 6d. in 1895, against £101 12s. 3d. in 1894, and the cost of each in-patient per day 5s. 8d., against 5s. 7d. in 1894.

